# A qualitative study of an undergraduate online emergency medicine education program at a teaching Hospital in Kampala, Uganda

**DOI:** 10.1186/s12909-022-03157-5

**Published:** 2022-02-08

**Authors:** Adeoluwa S. Ayoola, Peter C. Acker, Joseph Kalanzi, Matthew C. Strehlow, Joseph U. Becker, Jennifer A. Newberry

**Affiliations:** 1 Stanford University School of Medicine, Palo Alto, California USA; 2 Department of Emergency Medicine, Stanford University School of Medicine, Palo Alto, California, USA; 3 Department of Emergency Medicine, Makerere University College of Health Sciences, Kampala, Uganda

**Keywords:** Emergency medicine undergraduate education, Qualitative research, Medical education

## Abstract

**Background:**

Globally, half of all years of life lost is due to emergency medical conditions, with low- and middle-income countries (LMICs) facing a disproportionate burden of these conditions. There is an urgent need to train the future physicians in LMICs in the identification and stabilization of patients with emergency medical conditions. Little research focuses on the development of effective emergency medicine (EM) medical education resources in LMICs and the perspectives of the students themselves. One emerging tool is the use of electronic learning (e-learning) and blended learning courses. We aimed to understand Uganda medical trainees’ use of learning materials, perception of current e-learning resources, and perceived needs regarding EM skills acquisition during participation in an app-based EM course.

**Methods:**

We conducted semi-structured interviews and focus groups of medical students and EM residents. Participants were recruited using convenience sampling. All sessions were audio recorded and transcribed verbatim. The final codebook was approved by three separate investigators, transcripts were coded after reaching consensus by all members of the coding team, and coded data were thematically analyzed.

**Results:**

Twenty-six medical trainees were included in the study. Analysis of the transcripts revealed three major themes: [1] medical trainees want education in EM and actively seek EM training opportunities; [2] although the e-learning course supplements knowledge acquisition, medical students are most interested in hands-on EM-related training experiences; and [3] medical students want increased time with local physician educators that blended courses provide.

**Conclusions:**

Our findings show that while students lack access to structured EM education, they actively seek EM knowledge and practice experiences through self-identified, unstructured learning opportunities. Students value high quality, easily accessible EM education resources and employ e-learning resources to bridge gaps in their learning opportunities. However, students desire that these resources be complemented by in-person educational sessions and executed in collaboration with local EM experts who are able to contextualize materials, offer mentorship, and help students develop their interest in EM to continue the growth of the EM specialty.

**Supplementary Information:**

The online version contains supplementary material available at 10.1186/s12909-022-03157-5.

## Background

Globally, half of all years of life lost is due to emergency medical conditions, with low-income countries facing over four times the burden of emergency medical conditions compared to high-income countries [1]. To reduce morbidity and mortality, particularly in low- and middle-income countries (LMICs), it is essential to have a well-functioning emergency care system optimally staffed with providers trained in emergency medicine (EM) to improve the acute stabilization of ill or injured, undifferentiated patients [2, 3]. Unfortunately, due to constraints in both infrastructure and human resources, many LMICs are still working to develop cohesive emergency care systems and lack sufficient numbers of practitioners trained to provide quality emergency care. Therefore, there is an urgent need to train not only current providers, but the next generation of LMIC physicians and healthcare providers in the identification and management of emergency medical conditions [4, 5].

However, many factors limit the development of an EM healthcare workforce, including a lack of exposure to EM amongst medical students and limited access to specialized post-graduate EM training, along with concerns regarding cost and sustainability [5–7]. EM is still an emerging specialty in many LMICs; as of 2019, 42 of 135 LMICs recognized EM officially as a medical specialty [8]. Not surprisingly, a minority of medical schools in LMICs incorporate EM education into their standard curriculum. Similarly, although EM residency training programs are growing throughout Africa, their numbers and geographic distribution remain limited [9].

Consequently, it is imperative to find innovative, low cost means to fill the EM educational gap in LMICs medical education until a substantial cadre of emergency physicians and educators have been developed. Already, the African Federation of Emergency Medicine (AFEM) produces high quality, locally relevant training materials that are downloadable and available on-line for clinicians and students [10]. Online resources like those offered by AFEM, videos, and web-based modules all constitute electronic learning (e-learning) resources. App-based forms of e-learning further overcome the barriers students currently face in accessing EM curriculum by leveraging the increasingly widespread access to the internet and mobile devices - connecting experienced providers and educators from other countries to students in need [11, 12]. Blended learning courses integrate e-learning resources with traditional, in-person learning opportunities. E-learning courses have been shown to be readily implementable, equivalent to traditional learning methods in knowledge acquisition, well-received by students and educators, and are increasingly being used as a way to share knowledge in LMICs [11–14].

However, little research pertains to the development of effective EM undergraduate education resources in LMICs and the perspectives of the students on methods of EM learning and the increasing use of e-learning resources. Without understanding the current ways in which medical students who lack access to structured EM training acquire requisite knowledge and skills, it is difficult to best design EM educational interventions. Our study aims to understand the perspectives of Ugandan medical students and EM residents on needs and resource utilization for EM education, including the role of blended app-based EM courses to address a lack of EM focused curricular offerings. Although the study started from an evaluation of one such blended, app-based EM course, it used the course as a natural impetus for recruiting medical students and launching a broader conversation on the current state of EM education at their university, their academic needs, and their perception of e-learning resources, both in relation to the course and beyond it.

## Methods

We conducted semi-structured interviews and focus groups with medical trainees (undergraduate medical students and residents) at Makerere University School of Medicine (MUSM) and its partner hospital Mulago National Specialized Hospital in Kampala, Uganda. This study was approved by the School of Medicine Research and Ethics Committee at MUSM (#REC REF 2019–109) and the Stanford University School of Medicine Internal Review Board (IRB51200). We follow the Standards for Reporting Qualitative Research Guidelines [15].

### Setting

MUSM has a total enrollment of over 1000 undergraduate medical students at any given time, each going through a five-year curriculum to earn a Bachelor of Medicine and Bachelor of Surgery (MBChB). The curriculum is in transition from two to now three clinical years at Mulago. Situated in the capital city of Kampala, Mulago is the largest public hospital in Uganda with over 1500 beds. Its Accident and Emergency Unit serves as the emergency care delivery and triage focal point for the hospital and holds approximately 55 beds. The EM residency training program is a three-year program that was launched in 2017 and included eight EM residents in its first class, four in its second class, and four in its third class. The program was an interdisciplinary project that grew through support from anesthesia, surgery and critical care providers who were already providing routine emergency care at Mulago’s Accident and Emergency Unit. With the growth of the EM graduate program, there has gradually been an increased focus on EM in medical school. Currently, undergraduate EM education consists of first aid training for first year students and selected EM topics incorporated into the required anesthesia rotation in the clinical years. No EM-focused courses or rotations are a part of the formal curriculum to date.

### Blended, app-based foundations of emergency medicine course

The Foundations of Emergency Medicine course began as a collaboration between Stanford University School of Medicine and Makerere University School of Medicine in 2014. The goal of the course is to expose medical students to an EM-based approach to the acutely ill undifferentiated patient and selected foundational topics (see Additional File 1). It was designed to be accessible to students from a wide range of settings world-wide. Students access course material, delivered as online videos, via a mobile app built by the Digital Medic platform [16]. Students can download all video content using free Wi-Fi and can view it offline, avoiding data usage fees. As a complement to the online material, workshops lasting between 2 and 4 h are run by local instructors to discuss case scenarios, perform simulations, and practice hands-on skills.

In a prior prospective study [13], researchers found that students, whether from Stanford University or Makerere University, who enrolled in the app-based course had equivalent knowledge gains to those in a traditional classroom-based course. In 2019, the course was offered to all fifth-year medical students. The course was taught by three first-year EM residents over 3 days using the Digital Medic platform with condensed adjunct workshops.

### Data collection

We used a convenience sampling method to recruit medical school students at MUSM enrolled in the blended, app-based Foundations of Emergency Medicine elective course in the summer of 2019. The course is open to all medical students but is taken predominantly by student entering their fifth year. The medical school’s rising fifth year class had approximately 120 students and from this group, 73 medical students enrolled in the course. The course was free of charge and enrollment in our study had no impact on their course participation. Participants were recruited through the course’s WhatsApp group and in-class on the first session. Interested students could then reach out to the primary interviewer and an interview was scheduled based on their availability. Participation in the study was completely voluntary. Our methods employed both individual interviews and focus groups. The focus groups were conducted to elicit any further themes through a group perspective of the topic. To achieve these modes of data collection, participants were invited either to be interviewed individually or join a focus group based on their availability and preference.

We conducted semi-structured interviews and focus groups with fifth year medical students nearing completion of their undergraduate medical education, and first year EM residents to elicit a fuller perspective of undergraduate EM medical training at the Ugandan Medical School. The focus groups were performed at the end of the data collection period, which allowed prioritization of the initial interview questions based on emerging themes identified during the individual interviews. Additionally, focus groups were designed to uncover themes not elicited during individual interviews. As there were few residents available for participation, only individual resident interviews were conducted.

The semi-structured interview and focus group guides were developed based on the study aims, a literature review conducted prior to data collection, and feedback from prior iterations of the course. Specifically, we focused on the question of localization and contextualization of the course content. The interview guides were piloted with a MUSM graduate who had previously taken the course to ensure rigor and clarity of discussion questions and prompts (see Additional file 1). Guides were finalized after consultation with all authors. All interviews were performed in English, by one primary interviewer, AA. The guides asked main and probing questions in three domains:

1. Current access to and use of EM learning resources for medical trainees;
2. Needs for medical student EM knowledge and skills; and
3. Perspectives on the 2019 blended, app-based course.

All participants provided informed, written consent. All interviews and focus groups were recorded, with individual interviews and focus groups lasting approximately 1 hour. The recordings were transcribed verbatim and participants’ identities were redacted before analysis.

### Data analysis

The research team used Dedoose coding software [17] to store data, label codes, and confirm consensus on codes. Our analysis team included four different coders and data analyzers who looked at the data individually and jointly: authors AA, JB, JN, and PA. Author AA, who is a medical student at Stanford University, coded all interviews and focus groups. Authors JB, JN, and PA, who are academic physicians in EM at Stanford University, each coded a selection of transcripts, so that each transcript was ultimately reviewed by at least two coders. The codebook was started deductively from the three domains of the interview guides and updated inductively as coding progressed [18]. Following first round coding, all four coders deliberated until consensus was reached on all codes and the codebook was finalized. The research team used a conventional thematic analysis model to synthesize findings [18]. Themes were identified after the first round of coding, discussed with all coders, and constantly updated through the second round of coding until a consensus was reached.

## Results

We enrolled 26 medical trainees. We conducted 20 semi-structured individual interviews (13 medical students, 7 EM graduate residents) and two focus groups with three undergraduate medical students in each (Table 1). Of the student participants who were individually interviewed, 4 were interviewed prior to the start of the course while 9 were interviewed during or following the course. Focus groups were performed after the course had ended. None of the focus group participants was individually interviewed. Four of the seven residents interviewed were involved in the course as leaders, either past or present. All interviews with residents involved in the course occurred before the start of the course.

**Table 1:** Participant Characteristics

	5th year Medical Students	1st year EM Residents
Gender		
Male	13 (68%)	3 (43%)
Female	6 (32%)	4 (57%)
Interview type		
Individual	13 (68%)	7 (100%)
Focus group	6 (32%)	–

We identified three core themes through our analysis (Table 2): (1) medical trainees want education in EM and actively seek EM training opportunities; (2) although the e-learning course supplements knowledge acquisition, medical students are most interested in hands-on EM-related training experiences; and (3) medical students want increased time with local physician educators.

**Table 2:** Themes, Subthemes, and Quotes

Themes	Subthemes	Description and Quote
1. Ugandan medical trainees want education in EM and actively seek EM training opportunities.	Access to structured learning opportunities is limited and fragmented.	Formal educational opportunities: medical school classes, non-EM specific clinical rotations (anesthesia, general surgery), and class simulation sessions.
		Outside short courses: Basic Life Support, Advanced Trauma Life Support, Advanced Cardiac Life Support, community first aid responder course, and the World Health Organization’s Emergency Triage Assessment Treatment (pediatric) and Basic Emergency Care courses.
		“For example, me, throughout my medical school, I didn’t learn anything about emergency. I remember we had a trauma course, we learned how to intubate, but I was an intern and it didn’t make sense. So, really nothing much.” [Resident 5]
		“In our rotation schedule, there is really no direct provision about the emergency medicine rotation. Though we have an [accident and emergency] unit, that’s it. But there’s no course that allows us to go and learn. [Medical Student 13]
	Experiential learning opportunities are used to supplement theoretical knowledge with practical knowledge but are challenging to obtain.	Experiential learning on wards from mentors, peers, near peers, and nurses, and occasionally through practice or simulation.
		“… if you are lucky and you are able to find one of their residents performing that procedure, you stick around and you ask them questions and they answer you. And if they are kind enough, they can even let you perform, which is not so often, but yeah, you can observe them doing it.” [Focus Group 2]
	Trainees actively seek to fill gaps through unstructured, self-directed learning.	Learning resources mentioned by participants include: Internet Websites: Medscape, Life in the Fast Lane, Wikipedia Videos: YouTube, Kaplan videos Official Guidelines: AFEM handbook of Emergency Medicine Textbooks: Toronto Notes for Medical Students, Oxford Handbooks, Tintinalli’s Emergency Medicine Manual, Rosen’s Emergency Medicine: Concepts and Clinical Practice Others: Podcasts, Mobile apps
		“… I took my personal initiative and watch the videos on YouTube about how to interpret. Then I also google on Wikipedia on how to interpret those different radiographs.” [Medical Student 5]
		*“*Everyone has that own choice. And you go to YouTube, you’re going to look for what’s in your field.” [Medical Student 1]
2. Medical students are most interested in hands-on EM-related training experiences.	Students express an overall need for more EM clinical training in a safe, guided manner.	Students desired inclusion of skills labs, simulations, and guided rotations covering EM clinical skills.
		“if there was a real skills lab for us to practice some of those things. Yeah, on dummies, before we actually sutured the real people. It would be nice.” [Medical Student 2]
		“Basically, I should say they should put more of the teachers, people who help guide us when we are doing it practically, but also they should improve the skills lab and make it work in such that we practice these skills in the skills lab before we apply them on the patients.” [Medical Student 11]
3. Medical students want increased time with local physician educators.	Students most appreciate practice opportunities that are led in-person by experts and educators.	Students greatly appreciated the chance to put theoretical concepts into practice with expert guidance and hands-on learning in the midst of an e-learning course.
		“And also, the discussions were really helpful in a way that... they helped us. They took us step by step. Because it is different where you just sit and watch the videos because, when you actually reach to apply what you’ve watched, you may forget things and like jumble up everything. But they were there to guide us step by step, which I think was really very helpful. They really give us their time and we appreciate that they gave us their time to teach us.” [Focus Group 2]
	Students feel that contextualization by local EM instructors is necessary to increase relevance to their setting.	Trainees desired videos content, guidelines, or expert lectures portraying how to address situations in their clinical setting.
		“If a person who comes from a country where they have, per hospital, they have medical evacs, they have all the fancy and ideal things, come to teach us here, the input from our local faculty and things we can equally still do good emergency care to the patients. But in our setting, they put things into context a bit … I’m going to be able to save my Ugandan patient with whatever I have, so the local faculty bring that to light.” [Resident 3]
		“Yeah, actually these sessions, I think, were very tailored towards the Ugandan setting. They did not talk about anything out of the context. I realized that these cases, they were deriving them off head probably from their experiences working in the Ugandan setting. I think they really brought out our aspect here as in our setting. And yeah, it was very good.” [Medical Student 11]
	Students desire increased exposure and engagement with EM and EM role models.	Engagement in an EM clinical rotation, more interactions with EM physicians, and EM courses early and often in medical school.
		“I wish they can make it a discipline on its own like say a course week, a marked course, a part of the university program.” [Focus group 1]
		“So, if we have more Ugandan educators, I would want to have a Ugandan emergency physician with that kind of attitude that the external educators have. So, it will kind of encourage you as a Ugandan MMED if you have a Ugandan emergency physician. It kind of motivates you.” [Resident 7]

### Medical trainees want education in EM and actively seek EM training opportunities

All interviewees expressed that emergencies happen often and, especially in Uganda where EM is just blossoming as a department and specialty, EM training for all physicians should be a requirement: *“There are basic skills that I feel like every person who is having a medical undergraduate degree should have when it comes to helping patients and also emergencies that happen all the time. So, in my opinion, any individual should be able to at least manage some of those emergencies.”* [Focus Group 2].

Students described that they often encountered emergency situations during their clinical rotations but felt unconfident and unprepared. *“Well, I noticed a gap in our curriculum and that in the years that we’ve studied, in all the four years that we’ve studied medicine, we haven’t really like gone into detail on what to do in certain emergency situations and I didn’t think it was prudent to continue with the [medical school] course not knowing those things.”* [Medical Student 8].

Without formal EM training through their medical curriculum, Ugandan medical students instead receive this training through other avenues (Table 2). Their use of learning resources can be grouped into three categories: structured, experiential, and unstructured resources. Structured resources are defined as standardized curricular learning opportunities through formal training programs and medical school courses. Experiential resources are defined as hands-on learning from peers, attending physicians, and clinical exposures during hospital rotations. Unstructured resources are defined as informal avenues of self-directed learning gained from websites, apps, national and international guidelines, and other open learning resources.

#### Access to structured learning opportunities is limited and fragmented

Short, outside courses were in high demand but limited. Additionally, most structured courses described were run by non-Ugandan organizations. “*… I remember in undergraduate, we had ETAT, emergency triage and training for pediatric … Then the other one was actually some Basics of Emergency Care. It was another one-week training. I think it was pioneered by the anesthesia group. I don’t remember. There’s a couple of foreign faculty and all.”* [Resident 3].

#### Experiential learning opportunities are used to supplement theoretical knowledge with practical knowledge but are often challenging to obtain

Medical trainees reported that they learn EM predominantly through hands-on, guided training found on clinical rotations: *“Actually, most of the skills, we weren’t actually taught how to do it. We just learned on the ward with whoever’s there at the time.”* [Medical Student 8].

However, interviewees stressed that although experiential learning opportunities are their most important avenue for learning, these types of opportunities are limited in number. In describing these limitations, students identified the lack of a formal structure to clinical mentorship or teaching, a high student-to-teacher ratio, and a lack of protected time for physicians to teach. Students desired increased time for physicians to provide medical student education. *“Most of them [clinical educators] are good but most of them are always busy so if there’s someone who’s really there for you, you can learn a lot from them … Our challenge is time, I would just ask them to avail more time to students and come on wards to teach.”* [Medical Student 6].

#### Trainees actively seek to fill gaps through unstructured, self-directed learning

Often, structured courses and unstructured, experiential learning opportunities are not enough exposure to the EM training that students desire. Instead, students reported turning to unstructured, self-directed learning resources. These resources are typically free, easily accessible and often those suggested by peers or colleagues. The use of these resources greatly varied between trainees. While some sites of self-directed learning are validated through medical organizations and institutions, most were informal sites, hand-selected by the user. *“You know the internet is huge, you need to narrow down. Then, I didn’t have, like, a search engine. So, it was either Medscape or like Google...”* [Resident 7].

### Although the e-learning course supplements knowledge acquisition, medical students are most interested in hands-on EM-related training experiences with in-person instructors

#### Students express an overall need for more EM clinical training in a safe, guided manner

Safe in this case refers to psychologic safety with unevaluated learning that is free of consequences. Guided training refers to providing constructive feedback or opportunities to reapply learned skills. Students described that, often, *“learning on ward is opportunistic”* [Medical Student 2]. *“… many times people would come with massive pleural effusion that needed drainage, so I learned from the interns. I assisted but I’ve never actually done one myself. It helps that you see it done and then you’re taught but I wish it could be done on a dummy first before you actually try on any human being …*” [Focus Group 1].

### Medical students want increased time with local physician educators

Medical students found it particularly meaningful to interact with the Ugandan EM residents who taught the 2019 Foundations of EM course and expressed a desire for increased time with Ugandan physician educators.

#### Students most appreciate practice opportunities that are led in-person by experts and educators

Students valued the personalized guidance provided by instructors, the local contextualization instructors gave to the learning material, and the unique exposure to EM the in-person sessions provided. Though students felt that the course’s app-based video content effectively provided foundational information on EM and helped reduce barriers to accessing content, the in-person workshops with instructors were described as crucial as they allowed students a chance to put theoretical concepts into practice with expert guidance. One student expressed appreciation for the instructors who guided them step by step: *“… we always need a teacher or you might not do it as well as it should be done even if you watched a video. Because this is a person who is showing you how to hold a needle, how to turn a patient.”* [Medical Student 2].

Further, the enthusiasm of local instructors and their accessibility was a powerful motivator for students to explore EM further: *“Seeing instructors passionate about it, conjures a possibility in my head of ‘maybe I could do this’.”* [Medical Student 12].

#### Students feel that contextualization by local EM instructors is necessary to increase relevance to their clinical setting

Medical trainees expressed that the video content needed to be locally contextualized to practice in Uganda, even though the content was overall comprehensive, easy to understand, and engaging: *“The content was spot on, it’s just the setting and the localization...These people have been taught for five years to do Mulago system. If you can put that content and localize it, contextualize it to the setting in which they are used to, they would take up more of the knowledge and skills.”* [Resident 1].

We define contextualization here as EM educational content that focuses on common emergency cases encountered in the setting of use, the resources available and guidelines followed at local hospitals, and ways local providers improvise if recommended resources are not available. Students felt that this contextualization was provided during in-person workshops by local providers. For example, a focus group participant noted, *“I like the fact that [he] used to use local examples … you needed a cervical collar, but you don’t have it. Don’t have the luxury of having one, so what do you do? Use a blanket. I like those aspects.”* [Focus Group 1].

Trainees also identified the need for locally relevant learning material that includes expertise from local instructors in all aspects of their learning, as many learning resources were predominantly created abroad. Trainees desired videos, guidelines, or expert lectures portraying how to address situations in their clinical setting, based on local capacity and protocols. Many participants suggested the creation of additional videos locally, *“I wish in our Ugandan setting, we can have some Ugandan videos prepared by our lecturers here where they know real Ugandan cases ... we can always add the videos to our sessions and then to the simulations and then the skills lab. I think it’ll make us better than it has been before.”* [Focus Group 1].

#### Students desire increased exposure and engagement with EM and EM role models

Students experienced a positive increase in their perception of their competence in EM care. All participants said they would recommend the course to other medical students, and all felt the course had a positive impact on their perception of the importance of EM education. Even students who were not interested in entering the EM specialty described the strong importance of emergency care in every field of medicine: *“I’d strongly recommend it [the course] … reason being emergency care applies. You’ll come across these conditions. It’s needed almost everywhere, in every field.”* [Medical Student 11].

In particular, trainees noted wanting a core EM clinical rotation to be offered during medical school.

I would say it should be a core module. Part of the curriculum. Because it’s not reflected anywhere in the curriculum and it’s very important for any general doctor. Might not end up as emergency physicians but every doctor has to learn the general approach for approaching an acutely ill, undifferentiated patient. So, I wouldn’t just recommend it for any student, I would recommend that the university incorporates it in the curriculum. [Resident 1]

## Discussion

Our findings add to the literature on EM education and e-learning use in LMICs by incorporating the perspectives and needs of medical students. We found that Ugandan medical students were eager to have more training in EM because they saw EM as part of any physician’s practice in Uganda. Moreover, students desired an in-person component to their learning that provided access to local instructors, contextualization of e-learning educational content and overall exposure to EM and physician role models. As ever-increasing access to technology facilitates the sharing of online educational materials like the course presented in this study, it is important to understand how materials created in high-resourced settings can be successfully adapted for LMICs where resources and pathology may differ. Our study grows the needed discussion on contextualization and its necessity in international educational collaborations [19].

### Incorporate opportunities for EM training, practical clinical sessions, and emergency care management often and early in medical school

Our results showed that medical students recognize that emergencies occur across specialties and that it is necessary to gain knowledge and skills on how to approach emergencies, beginning with the undifferentiated patient, regardless of the career path a student plans to follow. Emergencies are anxiety provoking, and it takes not only exposure to allow students to translate their knowledge into action but also practice to build the skills necessary to address emergency presentations. In the current system at the school evaluated, students report relying heavily on unstructured teaching during clinical ward rotations and self-identified learning resources to create the foundation of their medical knowledge.

As noted in this study, students desire access to additional practical experiences, guided by experienced educators, early and often in medical school, to bridge this gap in EM training opportunities. This aligns with current research on clinical skills acquisition, as medical trainees express hands-on practice and learning from clinical seniors as important resources for informal learning [20–22]. The implementation of a required EM core rotation can create this learning space and strengthens medical student training, especially in the final clinical year [23, 24]. Our recommendation therefore is that medical schools include a core foundational EM rotation early in the medical school curriculum.

### Medical students need easily accessible, high-quality educational resources that e-learning courses provide but in-person instruction is still crucial

Our findings reveal a critical perception of the e-learning app-based course as a beneficial first step but inadequate alone to address the current gaps in EM education opportunities. Participants’ perception of the course’s quality and the positive impact it had on their learning aligns with the literature on the effectiveness of supplemental, e-learning and blended courses for teaching foundational medical principles [11–14].

However, our study reaffirms that in-person education is an important aspect of learning, even for students engaged with an e-learning course, and for mentorship. The in-person element allows for further details related to the content to be provided and is important for building mentorship opportunities early and often to support medical student learning [25, 26]. Based on the findings of this study, it is recommended that developers of e-learning courses support, engage, and collaborate with local educators to provide in-person learning and spur innovation in this area.

### Local contextualization of globally distributed educational materials can help increase student engagement

Current literature on medical e-learning tools do not fully explore the importance of contextualizing e-learning material to the local clinical setting [27–29]. To promote the success of e-learning courses, we strongly recommend local contextualization, utilizing the expertise and direct participation of local EM role models whenever possible. This contextualization may allow students to better apply the knowledge gained to their own practice settings and increases their engagement and trust in the course. We recommend that local EM experts be included in adapting courses created in high-income countries, they are not only better positioned to tailor the message to their student’s needs, but also to demonstrate the incredible knowledge and talent possessed by regional experts and its value to the learning process.

Students value the relationships built with their local instructors and can better imagine themselves as physicians providing emergency care when exposed to Ugandan EM physicians. Students’ appreciation of the exposure to local EM physicians during the course and their desire for increased mentorship during ward rotations shows the potential to create mentorship structures during clinical training and promote formal teaching opportunities for junior and senior attendings [30–32]. Access to local experts and mentors promotes the growth of the specialty itself. As existing literature shows, the personal role models students interact with in medical school greatly influence their specialty choice [33]. In a budding field like EM, in-person education by local EM experts is particularly important as these interactions offer a vital means through which students can gain exposure to the specialty and potentially drive interest in pursuing future training and eventual work in the field. This is especially relevant in Uganda as the medical education system begins to build a pipeline of qualified applicants to fill their new EM residency programs.

It is clear trainees in this study desire knowledge contextualized to Uganda’s setting, incorporating Uganda physicians, yet current literature on implementing EM training courses in LMICs has not put importance on the educators delivering the content [34]. Future work needs to be done to show how contextualized resources and implementation by local educators affect participants’ learning. It is also important to explore the degree of contextualization (regional, national, or continental) necessary to create an appropriately relevant educational material.

### Limitations

Our study is subject to selection bias in two ways. First, we only offered participation to students who had already clearly demonstrated their interest in EM by enrolling in the course. Second, we did not randomly sample from the class, but rather students self-selected to enroll. As a result, these students may be the most invested in gaining EM knowledge as compared to the average medical student at MUSM. We also note that several interviews were done during the course or following the course - this timing could have influenced students’ views on EM medical education and its importance. In addition, we include only a single academic institution in Uganda and EM topics and skills may be more or less fully integrated at other medical schools. Lastly, we acknowledge a pre-existing belief amongst the research team in the importance of EM education and training, which contributed to our design of the study and may have affected our interpretation. However, the importance of EM and its continued growth are supported by multiple national and international healthcare organizations.

## Conclusion

Students in Uganda and many LMICs lack access to structured EM education, which acts as a barrier to the development of effective emergency medical systems and the potential recruitment of future EM clinicians. Through an exploration of student perspectives, this study found that although Ugandan students actively seek EM knowledge and practice experiences, few formal, institutionalized opportunities exist which leads students to utilize self-identified, non-standard learning opportunities. Students identified e-learning programs as a means of sharing high quality EM education, while novel institutional EM programs continue to mature, however these programs alone are inadequate. Students desire e-learning resources to be complemented by in-person educational sessions, collaborating with and directly incorporating local EM experts who are able to further contextualize materials, offer mentorship, and help students develop their interest in EM to continue EM’s growth in these regions. Therefore, when designing and deploying e-learning EM educational content, it is important to consider the perspectives of the students who will use it, collaborate with professionals in the region regarding contextualization of the work whenever possible, and find ways to supplement online resources with in-person learning opportunities provided by local educators. Future studies should look at the effects of contextualized teaching on student engagement and knowledge acquisition.

## Supplementary Information

**Additional file 1.** Interview and focus group guides used in this study as well as a description of the blended app-based course used in our study.

## Data Availability

The datasets generated and/or analyzed during the current study are not publicly available but are available from the corresponding author on reasonable request.
