# Supplementary material for: A qualitative study of an undergraduate online emergency medicine education program at a teaching Hospital in Kampala, Uganda
Source: BMC Med Educ. 2022 Feb 8;22:84. doi: 10.1186/s12909-022-03157-5 (PMC8822823; doi:10.1186/s12909-022-03157-5)
Supplement: Supplementary file 1 — Additional file 1. Interview and focus group guides used in this study as well as a description of the blended app-based course used in our study. [file 12909_2022_3157_MOESM1_ESM.pdf]

<u>EM Uganda Course Interview Guide (Resident)</u>

Introduction

My name is Ade Ayoola and I am a medical student from Stanford University working with the Makerere Emergency Medicine Program this summer.

This interview is part of a research study that is looking at emergency medical education in Uganda. Participation is voluntary and refusing to participate involves no penalty. There are no foreseeable risks or benefits from participation in this study although this is the possibility of improving emergency medicine education in the future. If you agree to participate, the interview will last approximately 30 - 45mins. Everything you tell me will be used only for this research and will be kept confidential within the research team. The results of this research study may be presented at scientific or professional meetings or published in scientific journals. However, your identity will not be disclosed**.** You have the right to refuse to answer particular questions.
I have a consent form with more information about the research and who to contact about questions and your rights. You are welcome to read through it and ask any questions you may have. Please sign at the bottom if you're willing to participate.

Do you have any questions before we start?

Warm-up Questions

Tell me about how you got here to be an EM graduate resident?

- Did you do an internship or house officer position?

Opening Questions

How did you know you wanted to enter emergency medicine?
OR: What made you choose EM over other available specialties?

What motivated you to explore EM? How were you inspired to pursue the master's degree in Emed?

Tell me about the resources that helped you explore EM specialty.
Probe: Textbooks, classes, friends, TV shows?

How did you find these resources and how effective were they?
Probe: accessibility? Med school curriculum? Outside resources?

How involved are you with the emergency medicine course for undergraduate medical students?
If not very, skip next section and go to questions on educators/inspiration

Questions on Medical Student Course

[Section 1 – videos]
What do you think of the current online emergency medicine course videos?
Probe: speakers, topics/relevance, videos, format/cases, length, quality of video, quality of education

What do you think about the effectiveness of the current online emergency medicine course videos?
Probe: speakers, topics/relevance, applicability, format/cases, length, videos…

What are things the videos do well?
Probe: Accuracy. Quality. Understandability. Applicability.

If you could work with a team to improve the course videos, what would you change?
Probe: cases, instructors, context, length

Did anything stand out as less relevant, or less practical, to the practice of emergency medicine (either locally or as a physician more generally)?

Were there any concepts discussed that were taught in the videos that you did not feel would be practically applicable in your clinical setting? Which ones, and why did you feel that way?

[Section 2 – Discussions and workshops]

Question 2: how did the discussion sessions help students learn?
    Probe: applicability, quality of instruction, fit with overall course, length
    If negative: what aspects detracted from your learning?

What are things the discussions and workshops do well?
Probe: Accuracy. Quality. Understandability. Applicability.

If you were on a team to improve these sessions, what would you change to improve them?
Probe: cases, instructors, activities...

[*Section 3 - Overall effectiveness and impression*]
What was your impression of using the app-based platform to teach the material?
Probe: was it effective? Would you want to see leveraged otherwise?

How well do you think this course would have prepared you for the EM residency program?
Probe: Internship year or being a house resident?
    Would you have taken a course like this during medical school if it was available?

At the end of the course, how well prepared do you feel medical students are to assess and treat an acutely ill, undifferentiated patient?
Probe: effectiveness, applicability, information quality…

Would you recommend the course to current undergraduate medical students? Why or why not?

Questions on EM Medical Education - broad
Tell me about a teacher or educator that you felt you learned from most in medical school? What were their characteristics?

Who do you look to learn EM from? Why?
Probe: what are the characteristics, who are role models, how accessible

How much of your learning so far has been created and/or led by Uganda v foreign educators?

How important is it to have educational material like videos, lectures, textbooks, made by Ugandan educators to teach students in Uganda?

How might material that features Ugandan educators be viewed versus educational material made by and featuring foreign educators?

Tell me about how you see yourself as an educator as part of your role as emergency medicine resident? In the future?

What types of education have you received on how to be a medical educator? What sources of information have you found helpful in this area?

How do you balance your role as an educator with your roles as both a learner and health care provider?

What support would do you feel you need to grow as an emergency medicine educator?

Closing questions
If there was a two-day workshop on medical education, would people attend it? Would you attend it?
A part of ACEM.

If you were an undergraduate student what resources would you like to see to help you explore EM and be better prepared for the master's program?

<u>EM Uganda Course Interview Guide (Medical Student)</u>

Introduction
My name is Ade Ayoola and I am a medical student working with the emergency medicine department this summer.

[Consent script]
This interview is part of a research study that is looking at emergency medical education in Uganda. Participation is voluntary and refusing to participate involves no penalty. There are no foreseeable risks or benefits from participation in this study although this is the possibility of improving emergency medicine education in the future. If you agree to participate, the interview will last approximately 30 - 45mins. Everything you tell me will be used only for this research and will be kept confidential within the research team. The results of this research study may be presented at scientific or professional meetings or published in scientific journals. However, your identity will not be disclosed**.** You have the right to refuse to answer particular questions.
I have a consent form with more information about the research and who to contact about questions and your rights. You are welcome to read through it and ask any questions you may have. Please sign at the bottom if you're willing to participate.

Do you have any questions before we start?

What level are you in your education?

What clinical rotations have you completed?

Opening Questions
What made you interested in taking this course?
F/U: When did you first know you wanted to explore EM?

Tell me about the resources that were important in helping you explore EM as a specialty.
Probe: Professors, upperclassman who had taken the EM course, textbooks, classes, friends, TV shows?
    F/U: How accessible were these resources?

How did you find these resources and how effective were they?
Probe: accessibility? accuracy? Representative of EM in Uganda? Reputability?

Questions on current adjunct clinical skill development
What are some of the skills needed for clinical rotations that you are expected to know but were never formally taught in medical school curriculum?
e.g. reading a CXR, using an EKG, presenting to attendings, Lab skills, CT scans, ultrasound, surgical skills, handling fractures, splinting, suturing, bleeding…
How did you learn these skills?
Probe: Practicing on ward, outside classes, textbooks, upper classmates, shadowing physicians…
    Follow-up: tell me with that video did really well? Where was the video made from?

What were the challenges to accessing the knowledge to learn these skills?
Probe: not a formal part of medical school, not all in one place,

What resources would you like added to your education to help prepare you for clinical practice?
Probe: a course teaching these skills, videos showing the skills, practice sessions, textbook,

Questions on medical student course
[Section 1 – videos]
What do you think of the current online emergency medicine course videos?
Probe: speakers, topics/relevance, videos, format/cases, length, quality of video, quality of education

In what ways did these videos in improving your skills and knowledge? In what ways?
Probe: speakers, topics/relevance, applicability, format/cases, length, videos…

What are things the videos do well?
Probe: Accuracy. Quality. Understandability. Applicability.

If you could work with a team to improve the course videos, what would you change?
Probe: Cases, instructors, context, length

Did anything stand out as less relevant, or less practical, to your practice (either locally or as a medical student more generally)?

Were there any concepts discussed (techniques, interventions, etc.) that you learned about in the videos that you did not feel would be practically applicable in your clinical setting? Which ones, and why did you feel this way?

[Section 2 – Discussions and workshops]
What are the discussion sessions like?
How did the discussion sessions help you learn?
Probe: (consider the negative) applicability, quality of instruction, fit with overall course, length,
If negative: what aspects detracted from your learning?

What are things the discussions and workshops do well?
Probe: Accuracy. Quality. Understandability. Applicability.

If you could work with a team to improve the discussion sessions, what would you change?
Probe: cases. Instructors. Activities...

[Section 3 – Overall effectiveness and impression]
What was your impression of using the app-based platform to learn?
Probe: was it effective? Would you want to see it leveraged otherwise?

How prepared did you feel at the end of the course to assess and treat an acutely ill, undifferentiated patient?
Probe: effectiveness, applicability, long-term learning,

Tell us why you would or would not recommend the course to other undergraduate medical students.

Tell me how the course affects your interest in emergency medicine?

Questions on medical education – broad
Tell me about how you learn best.
Probe: skills, basic content, critical thinking... your personal experience. Most effective classes/teachers.
F/U: Can you describe that to me?

Tell me about a teacher or role model that you felt you learned from most in medical school.
OR: Thinking back on your educational journey, what were the attributes that made a great teacher?
Probe: preclinical curriculum, clinical settings,

What are some ways teachers can present information in a way that helps their students best learn?
Probe: more cases? Videos?

How much of your education is taught by Ugandan vs. Foreign educators?

How important is it to have educational material like videos, lectures, textbooks, made by Ugandan educators to teach students in Uganda?

Would you like to see more Ugandan-made material?
Probe: videos, textbooks, clinical case practice…

How is the teaching by Ugandan educators viewed by students? Do you hold this view as well?

How is teaching by foreign educators viewed? Do you hold this view as well?

If there was one thing you would add to the medical curriculum to help you be a better physician, what would you add?

Closing questions

What medical specialty are you considering after medical school?

What most influences/influenced your choice in medical specialties?

How much teaching do you do to your peers and classes below you?

Back-up questions:
What is the teaching style of most Ugandan professors? How effective do you think it is at teaching students the material?
Probe: preclinical and clinical material

How much of your education has been led by foreign educators?
Probe/FU: Have you had guest speakers? (involved in main curriculum or teaching adjunct classes? Does it matter which they talk about?)

What is the teaching style of foreign educators? How well are they able to relay the information to students?

Focus Group Interview Guide

Introduction

Thank you all for coming today. My name is Ade Ayoola and I am a medical student working with the emergency medicine department this summer.

This group discussion is part of a research study that is looking at ways to improve the medical education courses for medical students and how certain changes will be perceived by the participants of the course. I am holding discussions with medical students in this online emergency medicine course to learn what they think of the course and recent changes to it.

[Consent script]
Today's discussion will take about an hour. Participation is voluntary, you are free to leave if you don't want to participate and refusing to participate involves no penalty. However, we greatly value your opinions and experiences and hope you will stay. There are no foreseeable risks or benefits from participation in this study although this is the possibility of improving emergency medicine education for medical students in the future. If you agree to participate, I will be asking you all a couple questions about your views and experiences. There are no right or wrong answers so please feel comfortable saying what you really think. We want to hear as many perspectives as possible so feel free to disagree with others and share your views. I will be taking notes while we talk so that I don't miss anything but in order to make sure we get down the views expressed today I want to record our conversation. Is that okay? Everything you tell me will be used only for this research and will be kept confidential within the research team. Your name will not be used in presenting the results and what you say will not be linked to you in any way. I have a consent form with more information about the research and who to contact about questions and your rights. You are welcome to read through it and ask any questions you may have. Please sign at the bottom if you're willing to participate.

Do you have any questions before we start?

Opening Question:
As an introduction, let's go around and say what year you are currently in your studies and what you might be interested in doing after.

Introductory Questions (may skip in the interest of time if rapport is already present):
What are some activities students do over their break?

Transition Question:
Why did you decide to take this EM course?
Probe: interested in EM? Past exposure to EM?

What resources do students often use to explore different specialties.
Probe: Professors, upperclassman who had taken a short course, textbooks, classes, friends, TV shows?

F/U: How accessible were these resources?

How effective were they?
Probe: accessibility? accuracy? Representative of EM in Uganda?

Questions on current adjunct clinical skill development
What were skills that you needed for clinical rotations but were not formally taught to you?

How do students usually learn the clinical skills they need to use while on ward?
Probe: Practicing on ward, outside classes, textbooks, upper classmates, shadowing physicians…
Follow-up: tell me with that video did really well? Where was the video made from?

What were the challenges to accessing the knowledge to learn these skills?
Probe: not a formal part of medical school, not all in one place,

What resources would you like added to your education to help prepare you for clinical practice?
Probe: a course teaching these skills, videos showing the skills, practice sessions, textbook,

Key Questions:
[Section 1 – videos]
What do you all as students think about the current online emergency medicine course videos?
Probe: speakers, topics/relevance, applicability, format/cases, length…

What are things the videos do well?
Probe: Accuracy. Quality. Understandability. Applicability.

If you could work with a team to improve the course videos, what would you change?
Probe: Cases, instructors, context, length

[Section 2 – Discussions and workshops]
What do you all think about the discussion sessions and workshops?
Probe: applicability, quality of instruction, fit with overall course

What are things the discussions and workshops do well?
Probe: Accuracy. Quality. Understandability. Applicability.

If you could work with a team to improve the discussion sessions, what would you change?
Probe: cases. Instructors. Activities...

[Section 3 – Overall effectiveness and impression]
What was your impression of using the app-based platform to learn?
Probe: was it effective? Would you want to see it leveraged otherwise?

How prepared did you feel at the end of the course to assess and treat an acutely ill, undifferentiated patient?

Probe: effectiveness, applicability, long-term learning,

Tell us why you would or would not recommend the course to other undergraduate medical students.

How did the course affect your interest in emergency medicine?

Closing questions
Summary of themes discussed
OR: Out of all the things we have discussed, what aspect of an educational course do you believe makes it more effective, engaging or motivational?

Thank you for your time. Do you have any questions for me?

If there is time:
Questions on medical education – broad
Tell me about a teacher or role model that students learned from most in medical school.
OR: Thinking back on your educational journey, what were the attributes that made a great teacher?
Probe: preclinical curriculum, clinical settings,

What are ways your teachers present information that you feel most help their students learn?
Probe: more cases? Videos?

How much of your education so far has been led by Ugandan instructors and how much has been led by instructors from other countries?

How is the teaching by Ugandan educators viewed by students? Do you hold this view as well?

How is teaching by foreign educators viewed? Do you hold this view as well?

It sounds like you have to use a lot of learning resources from elsewhere (videos, blogs...), would you like to see more Ugandan-made material?
Probe: videos, textbooks, clinical case practice…

How important is it to have Ugandan physician teaching medical students?
Probe: Preclinical, clinical

Foundations of Emergency Medicine Course Collaboration

The Foundations of Emergency Medicine course began as a collaboration between Stanford University School of Medicine and Makerere University School of Medicine in 2014. The goal of the course is to expose medical students to an EM-based approach to the acutely ill undifferentiated patient and selected foundational topics. It was designed to be accessible to students from a wide range of settings world-wide. Students can download all video content using free Wi-Fi and can view it offline, avoiding data usage fees. As a complement to the online material, workshops lasting between 2-4 hours are run by local instructors to discuss case scenarios, perform simulations, and practice hands-on skills.

**Module**	**Topic**	**Monday-Friday Asynchronous Learning Items**	**Weekend Group Learning Items**	**Group Learning Skills**
1	Approach to assessing the undifferentiated patient	Pre-Course Survey<br>Pre-Test<br>Approach to the Undifferentiated Patient Videos 1+2	Initial Patient Assessment Case Discussion	Application of systematic patient assessment
2	Basic life-saving interventions	Basic Life Support and Airway Management Videos 1-5<br>Case Discussion Video<br>Expert Analysis Video	Airway Opening Case Discussion<br>Unresponsive/Pulse Patient Case Discussion	Head tilt/chin lift, jaw thrust, bag mask ventilation, CPR, choking treatments
3	Shock	Shock Videos 1-3<br>Expert Analysis Videos 1-3	Distributive Shock Case Discussion<br>Cardiogenic Shock Case Discussion	Shock exam/assessment, vascular access strategies
4	Trauma assessment and skills	Initial Management of Trauma Patients Videos 1-4<br>Case Discussion Video<br>Expert Analysis	Trauma Assessment Case Discussion	Application of primary and secondary survey, extremity hemorrhage control
5	Basic wound management	Basic Wound Management Videos 1-5<br>Post-Course Survey<br>Post-Test	Wound Management Case Discussion	Suturing workshop
